# 2013 Update: Hopkins Lupus Cohort

**DOI:** 10.1007/s11926-013-0360-0

**Published:** 2013-07-26

**Authors:** Monthida Fangtham, Michelle Petri

**Affiliations:** Division of Rheumatology, Johns Hopkins University School of Medicine, 1830 East Monument St Suite 7500, Baltimore, MD 21205 USA

**Keywords:** Systemic lupus erythematosus, SLE, Hopkins lupus cohort, Prednisone, Organ damage, Hydroxychloroquine, Thrombosis, Lupus nephritis, Lupus anticoagulant, Cardiovascular disease, Cognitive impairment, SLE myelitis, Small fiber neuropathy, Vitamin D, Flares

## Abstract

The Hopkins lupus cohort is a longitudinal cohort study of over 2,000 systemic lupus erythematosus (SLE) patients, who are seen quarterly. This review covers ten important clinically-relevant studies of the cohort. These studies include the function of prednisone in atherosclerosis and thrombosis, the preventive function of hydroxychloroquine, new insights into rare neurological manifestations, and treatment of flares with bursts of steroids rather than maintenance steroids.

## Introduction

SLE clinical research should, ideally, provide information for diagnosis, treatment, and management of the disease. This is the purpose of the Hopkins lupus cohort, which started in 1987 and now includes over 2,000 SLE patients followed by one provider. The study design is to see patients quarterly, or more often if warranted by disease activity or complications. Quarterly follow-up has been confirmed as an ideal interval to avoid missing new manifestations of SLE [1]. The Caucasian and African–American ethnic mix has enabled us to address some important ethnic aspects of SLE, but the relative lack of Asian and Hispanic American participants means our findings may not generalize to all SLE patients. From the start the study has used the physician global assessment, the SLE disease activity index [2], and the lupus activity index (visual analogue scales for each organ system) [3]. This has enabled us to analyze both global and organ-specific SLE activity.

This review will highlight ten findings from the Hopkins lupus cohort that have immediate relevance to clinical practice.

## Prednisone and Organ Damage

SLE is managed with corticosteroids, because they work, work for most organs, and work quickly. The long-term harm caused by corticosteroids is now recognized, and “steroid sparing” is an FDA indication for new SLE treatment. The Hopkins lupus cohort has contributed to knowledge about corticosteroid toxicity in SLE in several important ways.

First, we have re-defined the cut-off for “high-dose prednisone”: rather than 20, 40, or 60 mg, it is any dose above 6 mg. Above 6 mg, risk of later organ damage increases by 50 %; higher doses are associated with even higher risk (Table 1) [4•]. Prednisone doses greater than 18 mg daily, which are commonly used in lupus nephritis regimens, increase later organ damage 2.5-fold. The risk of corticosteroid-induced organ damage is recognized as unacceptable by both rheumatologists and nephrologists. Elizabeth Lightstone, for example, has pioneered corticosteroid-free lupus nephritis regimens [5].

**Table 1:** Hazard ratio of organ damage (*n* = 141) by cumulative average dose of prednisone

Average dose prednisone	Hazard ratio^a^ for organ damage	95 % Confidence interval
>0–6 mg day^−1^	1.16	0.54, 2.50
>6–12 mg day^−1^	1.50	0.58, 3.88
>12–18 mg day^−1^	1.64	0.58, 4.69
>18 mg day^−1^	2.51	0.87, 7.27

^a^Adjusted for confounding by indication caused by SLE disease activity

Reprinted from Thamer et al. [4•], with permission from The Journal of Rheumatology

Second, the method of corticosteroid administration is important. Intravenous methylprednisolone seems to be safest [6]; however, it does lead to increased risk of cognitive impairment. For infants, corticosteroids are associated with cognitive impairment [7, 8]. For adults, intravenous methylprednisolone pulse therapy is routinely used for central nervous system (CNS) SLE, meaning resulting cognitive impairment could be caused by bias of indication.

High daily oral prednisone is associated with cataracts and with avascular necrosis of bone. The “threshold” effect of prednisone on avascular necrosis is well known. Even low-dose ongoing oral prednisone is not safe, being associated with cataracts, osteoporotic fracture, and cardiovascular events. Osteoporotic fractures are the most frequent musculoskeletal damage associated with SLE, and there is no safe dose of prednisone in terms of bone density. Because SLE patients are usually maintained on corticosteroids, there are no “corticosteroid holidays” that might enable recovery of bone density. SLE osteoporosis is multi-factorial, and is not just caused by prednisone; vitamin D deficiency and wide use of proton-pump inhibitors also contribute to increased risk.

## Hydroxychloroquine and Lupus Nephritis

Lupus nephritis remains a major unmet need for SLE patients. Current treatment regimens enable only a minority of patients to achieve complete renal response at six months. Adjunctive therapy, including ACE inhibitors and angiotensin receptor blockers, has become accepted. Both the ACR [9] and EULAR SLE nephritis guidelines [10] accept hydroxychloroquine as adjunctive therapy.

Our prospective database has revealed that hydroxychloroquine increases complete renal response with mycophenolate mofetil (MMF) therapy for membranous lupus nephritis. Of patients on hydroxychloroquine, 64 % achieved remission within a year, compared with only 22 % of those not on hydroxychloroquine (*p* = 0.036 on the basis of a log-rank test) [11]. These data are supported by several other studies. In a follow-up study of the Canadian hydroxychloroquine study group, in which randomization and blinding were not maintained, nephritis flare incidence was reduced by 74 % for those continued on hydroxychloroquine but did not reach statistical significance (*p* = 0.25) [12]. Fessler et al. have reported that hydroxychloroquine may delay or prevent renal damage [13].

Because SLE renal failure has not been reduced, use of “multi-target” therapy should be routine. However, hydroxychloroquine dose must be reduced for patients with renal insufficiency, and after five years’ use yearly monitoring for retinopathy is necessary.

## Hydroxychloroquine and Thrombosis

Several hematologic and immunologic mechanisms may contribute to hydroxychloroquine’s antithrombotic effect [14]. Hydroxychloroquine significantly diminishes thrombus size in a murine model of antiphospholipid antibody-induced thrombosis [15]. In the Hopkins lupus cohort we proved that hydroxychloroquine use reduced thrombosis, particularly for antiphospholipid-positive patients. Prednisone use increased thrombosis (even in multivariate models adjusted for disease activity). Aspirin use was not protective, instead increasing the risk of thrombosis. This is probably a bias of indication, because it is routinely prescribed to SLE patients who have antiphospholipid antibodies. Use of NSAIDs (predominantly naproxen) was protective against thrombosis, probably because of naproxen’s anti-platelet effect [16].

## Lupus Anticoagulant is the Most Important Antiphospholipid Antibody in SLE

Antiphospholipid antibodies are frequently observed in SLE, with approximately 10 % of SLE patients eventually developing antiphospholipid syndrome. Lupus anticoagulant measured by use of the dilute Russell viper venom time (dRVVT) is more strongly associated with SLE thrombotic events, miscarriage, and pulmonary hypertension than anti-cardiolipin measured by use of antibody assay (*p* < 0.01) [17]. In the Hopkins lupus cohort, lupus anticoagulant is a better predictor of risk of venous thrombosis than anti-cardiolipin. At 20-year follow-up there is a 42 % chance of deep venous thrombosis for SLE patients with a positive lupus anticoagulant measured by use of dRVVT [18]. Lupus anticoagulant is the only antiphospholipid antibody associated with myocardial infarction. Neither lupus anticoagulant nor anti-cardiolipin is associated with atherosclerosis [19].

In the PROMISSE study, lupus anticoagulant was the only antiphospholipid predictor of adverse pregnancy outcome for aPL-associated pregnancies after 12 weeks’ gestation [20]. In the Hopkins lupus cohort, the strongest predictor of pregnancy loss for SLE patients is lupus anticoagulant in the first trimester, measured by use of dRVVT testing. These data suggest that treatment of lupus anticoagulant should be considered even when there is no history of miscarriage [21].

## Cardiovascular Disease in SLE

Cardiovascular disease is the leading cause of SLE morbidity and mortality. Manzi et al. reported that coronary artery events were 50-fold higher for female SLE patients aged 35–44 years than for the Framingham population [22]. In the Hopkins lupus cohort, the overall risk of cardiovascular event associated with SLE is 2.66 times higher than for the Framingham population, after controlling for traditional cardiovascular risk factors [23••].

It has previously been reported for the Hopkins lupus cohort that age, hypertension, hyperlipidemia, obesity, and prednisone use are important risk factors for coronary artery disease in SLE [24]. Age and obesity are associated with coronary calcium, a surrogate measure of coronary atherosclerosis [25]. Our recent analysis confirmed that traditional cardiovascular risk factors including age, sex, mean systolic blood pressure, serum cholesterol, lupus anticoagulant, current corticosteroid dose, and presence of anti-dsDNA are associated with SLE cardiovascular events [23••], and has identified several SLE-specific factors associated with cardiovascular risk: low complement, SLEDAI disease activity, and lupus anticoagulant [23••].

Finally, treatment has an effect. Prednisone increases cardiovascular risk, both directly and, via increasing traditional cardiovascular risk factors (hypertension, hyperlipidemia, obesity, diabetes), indirectly [24]. Hydroxychloroquine may reduce the risk. The multivariate model is summarized in Table 2.

**Table 2:** Multivariable model of predictor and cardiovascular event rates, 1987–2010

Predictor	Rate ratio	95 % CI	*P* Value
Age per 10 years	1.63	1.421, 1.88	<0.0001
Male sex	1.56	1.01, 2.67	0.046
Year before 1993	1.64	0.99, 2.63	0.053
Mean systolic blood pressure^a^	1.17	1.02, 1.35	0.022
Mean serum cholesterol^a^	1.04	1.01, 1.08	0.018
Diabetes mellitus	1.52	0.99, 2.33	0.057
SELENA-SLEDAI	1.05	1.00, 1.11	0.062
Anti-dsDNA	1.56	1.05, 2.31	0.026
Serum creatinine, mg/dL			
- <1.0	1.00	Referent	Referent
- 1.0–1.19	1.64	1.07, 2.50	0.023
- ≥1.2	1.15	0.72, 1.85	0.56
Low hematocrit	1.18	0.82, 1.69	0.38
History of hemolytic anemia	1.28	0.79, 2.09	0.32
History of lupus anticoagulant	1.74	1.22, 2.47	0.0021
Current corticosteroid dose, mg/day			
- 0	1.00	Referent	Referent
- 1–9	1.01	0.63, 1.60	0.98
- 10–19	1.47	0.90, 2.38	0.12
- ≥20	2.54	1.44, 4.48	0.0013
Hydroxychloroquine (in past six consecutive months)	0.77	0.54, 1.12	0.17

Abbreviations: CI, confidence interval; dsDNA, double stranded DNA; SELENA, safety of estrogens in lupus erythematosus national assessment; SLE, systemic lupus erythematosus; SLEDAI, systemic lupus erythematosus disease activity index instrument score

^a^Mean during previous cohort participation

Reprinted from Magder and Petri [23••], with permission from Oxford University Press

## Cognitive Impairment in Systemic Lupus Erythematosus

Brey and colleagues drew attention to the importance of SLE-associated cognitive impairment in their SALUD study, which found that ten years after SLE diagnosis only 21 % of patients had normal cognitive function [26]. Wider use of cognitive testing, and repeated testing of cognitive function over time, was enabled by use of automated neuropsychological assessment metrics (ANAM) tests [27, 28]. Several aspects of cognitive impairment are tested by this repeatable, computer-based, neuropsychological group of tests, which generates a new test for each repeated use to help reduce the learning effect.

The “Brain CONECTIONS” (brain imaging and cognitive function in SLE) study was the first multicenter United States observational study of SLE cognitive functioning and brain imaging. The SLE patients in this study were newly diagnosed, had mild disease activity and damage index scores, and were not selected for neuropsychiatric manifestation. It was hypothesized that cognitive impairment would usually accrue over time; instead SLE patients had lower ANAM scores than normal controls in four of nine ANAM subtests administered at baseline. Cognitive impairment was not associated with corticosteroid use [29]. Moreover, 25 % of the newly diagnosed SLE patients had anatomic brain abnormalities visible on MRI, with more having cerebral atrophy than white-matter lesions [30], suggesting SLE cognitive impairment begins early in the disease.

Antiphospholipid antibodies were not associated with cognitive impairment of newly diagnosed SLE patients. However, the “Brain CONECTIONS” study revealed that depression was associated with significantly poorer cognitive function in several cognitive domains [31]. Depression was identified by use of a questionnaire rather than via physician recognition. A third important finding was that cognitive impairment either remained stable or improved over time. This is encouraging for SLE patients and their physicians. Finally, brain positron emission tomography (PET) scans found CNS white matter inflammation in newly diagnosed SLE patients, indicating a possible mechanism of SLE cognitive impairment [32].

## New Insights into Neurological SLE: SLE Myelitis and Small-Fiber Neuropathy

SLE myelitis is one of the most devastating nervous system manifestations of SLE. The Hopkins lupus cohort has contributed to two important discoveries regarding SLE myelitis. First, it is a longitudinal rather than transverse myelitis, which is one of the reasons it is so devastating. Second, SLE myelitis has two distinct clinical patterns: gray-matter and white-matter myelitis [33]. Patients with gray-matter myelitis often had a prodrome of fever and urinary retention at initial presentation, but the importance of this was usually missed (Table 3). These patients deteriorated rapidly, reaching a clinical nadir within 6 h; most were paraplegic at nadir and never improved. Gray-matter myelitis usually occurred in the context of SLE disease activity measured via SLEDAI scores. The cerebrospinal fluid (CSF) profile had striking inflammatory features resembling those of bacterial meningitis, including high CSF white blood cell counts, neutrophilic pleocytosis, high protein levels, and low CSF glucose levels. MRI results were more likely to reveal spinal cord swelling and enhancement. A longitudinally extensive pattern of inflammation involving at least three vertebral segments was observed for 91.7 % of gray-matter myelitis, compared with 73.9 % of white-matter myelitis. Patients with gray-matter myelitis usually had more disability, and were resistant to treatment despite receiving intensive immunotherapy; these patients probably already had irreversible injury when immunosuppressive treatment was initiated.

**Table 3:** Characteristics of myelitis associated with systemic lupus erythematosus

	Gray matter	White matter
Prodromal symptoms	Fever, urinary retention	
Neurological manifestations	Flaccidity, hyporeflexic	Spasticity, hyperreflexic
Disease course	Monophasic	Polyphasic
Disease activity	SLEDAI 9.8	SLEDAI 2
CSF finding	CSF ≈ bacterial meningitis	
Autoantibodies		Neuromyelitis optica IgG (NMO) Antiphospholipid antibodies

Data from Birnbaum et al. [33]

White-matter myelitis was characterized by upper-motor neuron spasticity and hyperreflexia at the time of the initial attack. Presentation was less severe than for gray matter myelitis, with slower progression. White-matter myelitis occurred when SLE disease activity was absent or was less severe, and prodromal features were usually absent. White-matter SLE myelitis is closely related to the neuromyelitis optica disease spectrum, i.e. optic neuritis, relapsing disease, and neuromyelitis optica IgG autoantibody. Neuromyelitis optica IgG autoantibody, anti-Ro antibodies, and antiphospholipid antibodies, including lupus anticoagulant, were associated with white-matter SLE myelitis. A longitudinally extensive pattern of inflammation and spinal cord swelling was observed, but post-gadolinium enhancement was more common in white-matter than gray-matter myelitis. White-matter myelitis usually had a polyphasic course, with related disability the result of recurrent attacks rather than of initial disease severity.

The frequency of SLE peripheral neuropathy documented by electrophysiology has been estimated to be approximately 30 % [34, 35]. It is now recognized, however, that SLE patients can have a small-fiber neuropathy which is electrophysiologically silent and may be missed. Small-fiber neuropathy can be distal, but may also be proximal if the proximal dorsal root ganglion is involved. This can result in the correct diagnosis being missed. Evaluation is by means of skin biopsy, immunostained to detect intraepidermal nerve fibers. Small-fiber neuropathy was not included in the ACR neuropsychiatric case definitions [36], but is now recognized as a common cause of SLE peripheral neuropathy [37].

## Vitamin D

Vitamin D deficiency has been associated with autoimmune diseases, including multiple sclerosis, type 1 diabetes mellitus, inflammatory bowel disease, rheumatoid arthritis, and SLE [38]. Vitamin D deficiency is more common for SLE patients than for matched controls. A Toronto study found that SLE patients’ vitamin D deficiency was related to season, cumulative glucocorticoid dose, and serum creatinine [39].

Vitamin D level may affect pathogenesis and disease course of SLE via immunomodulatory effects. Vitamin D also has immunomodulatory functions in murine models, and early vitamin D supplementation for lupus mice resulted in fewer dermatological lesions, less proteinuria, and lower anti-DNA titers [40]. Although the association is not always observed [41], several cross-sectional studies have reported an inverse relationship between vitamin D level and human SLE activity [42–46]. Our prospective study of 1,006 SLE patients found a modest but statistically significant improvement in SLE disease activity, assessed by use of the physician’s global assessment score or the SELENA–SLEDAI, associated with higher serum 25(OH)D [47]. For SLE patients with 25(OH)D <40 ng/mL, a 20 ng/mL increase in 25(OH)D was associated with a 21 % reduction in their probability ratio for a high disease activity score. No additional benefit was observed for those with serum 25(OH)D of 40 ng/mL or more. We also found a statistically-significant relationship between changes to serum 25(OH)D and improved urine protein-to-creatinine ratio. A 20 ng/mL increase in 25(OH)D was associated with a 15 % reduction in the probability of having clinically important proteinuria (urine protein-to-creatinine ratio >0.5). We therefore now use 40 ng/mL as our target 25(OH)D level for routine clinical care.

## Effect of Urine-Protein-to-Creatinine Ratio

SLE proteinuria is both albuminuria and globulinuria: because the urine dipstick only measures albumin, it can underestimate the true extent of proteinuria. Sieder et al. compared three urine dipstick assays used to detect proteinuria with the 24-hour protein-to-creatinine ratios of patients with lupus nephritis and found high variability [48]. This suggests the urine dipstick is not an adequate proteinuria screening tool for patients with lupus nephritis.

Instead, we have helped to pioneer use of “spot” urine-protein-to-creatinine ratios at every visit as the best method for early detection (and therefore early biopsy) of lupus nephritis. For clinical research trials, the protein-to-creatinine ratio obtained via 24-hour urine collection is best practice. For clinical care, however, the spot urine-protein-to-creatinine ratio correlates highly with 24-hour total urine protein and with 24-hour urine-protein-to-creatinine ratio [49].

Because proteinuria varies throughout the day, larger timed samples are preferable, if possible. The 12-hour overnight interval can provide a precise and accurate representation of 24-hour proteinuria, without the inconvenience of 24-hour collection [50].

## Treatment of Mild to Moderate Flare

Systemic lupus erythematosus (SLE) has two main patterns: “flare” and constant activity [51]. Corticosteroids are the usual therapy for acute flare, with administration method and dosage schedule primarily dependent on severity and organ involvement. A short course of oral corticosteroids can be used to treat many mild to moderate lupus flares; often, however, oral prednisone initiated to treat a flare is not reduced until the next visit, which may be months in the future. Noncompliance can reduce corticosteroid effectiveness; intramuscular triamcinolone can avoid this problem.

The FLOAT trial compared a one-week course of oral methylprednisolone (a “Medrol Dose Pack”) with 100 mg intramuscular triamcinolone [52]. This trial enrolled patients with mild to moderate SLE flares, most of which were cutaneous, arthritis, and serositis flares. SLE patients were randomized to Medrol Dose Pack or triamcinolone injection. Follow-up was after one day and one, two, three, and four weeks. Follow-up was not blinded; patients were graded on the basis of whether they were better using the SF-36. Both treatments worked. Triamcinolone patients reported greater numerical response at day one and week two, suggesting a slightly faster onset; there was no difference, however, at week four.

The FLOAT trial suggests that triamcinolone injection may be the preferred therapy for mild to moderate SLE flare. Because of the potential for focal lipodystrophy, the injection can be given in the buttock only. Patients can be given the option of the Medrol Dose Pack, especially if they cannot attend for a visit to receive an injection. Importantly, each therapy avoids the problem of raising the daily dose of prednisone over weeks to months and the resulting increase in corticosteroid toxicity.

## Conclusions

The Hopkins lupus cohort has revealed both problems with current treatment approaches (toxicity of prednisone) and the benefit of hydroxychloroquine. Early detection of lupus nephritis and coronary artery disease and use of multi-target approaches are important future priorities.
